# Detection of Cancer with Serum miRNAs on an Oligonucleotide Microarray

**DOI:** 10.1371/journal.pone.0006229

**Published:** 2009-07-14

**Authors:** Michael J. Lodes, Marcelo Caraballo, Dominic Suciu, Sandra Munro, Amit Kumar, Brooke Anderson

**Affiliations:** CombiMatrix Corporation, Mukilteo, Washington, United States of America; Cinvestav, Mexico

## Abstract

Micro RNAs (miRNAs) are a class of small, non-coding RNA species that play critical roles throughout cellular development and regulation. miRNA expression patterns taken from various tissue types often point to the cellular lineage of an individual tissue type, thereby being a more invariant hallmark of tissue type. Recent work has shown that these miRNA expression patterns can be used to classify tumor cells, and that this classification can be more accurate than the classification achieved by using messenger RNA gene expression patterns. One aspect of miRNA biogenesis that makes them particularly attractive as a biomarker is the fact that they are maintained in a protected state in serum and plasma, thus allowing the detection of miRNA expression patterns directly from serum. This study is focused on the evaluation of miRNA expression patterns in human serum for five types of human cancer, prostate, colon, ovarian, breast and lung, using a pan-human microRNA, high density microarray. This microarray platform enables the simultaneous analysis of all human microRNAs by either fluorescent or electrochemical signals, and can be easily redesigned to include newly identified miRNAs. We show that sufficient miRNAs are present in one milliliter of serum to detect miRNA expression patterns, without the need for amplification techniques. In addition, we are able to use these expression patterns to correctly discriminate between normal and cancer patient samples.

## Introduction

MicroRNAs (miRNA) are single-stranded RNA molecules of about 21–23 nucleotides in length, which function in the regulation of gene expression. miRNAs are expressed as part of primary transcripts in the form of hairpins with signals for dsRNA-specific nuclease cleavage by the ribonuclease Drosha in combination with an RNA-binding protein. After the precursor miRNA is released as an approximately 70 nt RNA, it is transported from the nucleus to the cytoplasm by Exportin-5, and then is cleaved by Dicer RNase III to form a double-stranded RNA. Dicer initiates the formation of the RNA-induced silencing complex (RISC), which is responsible for the gene silencing observed due to miRNA expression and RNA interference [1], [2], [3].

MicroRNAs have been found in tissues and also in serum and plasma, and other body fluids, in a stable form that is protected from endogenous RNase activity (in association with RISC, either free in blood or in exosomes (endosome-derived organelles)). Studies by Lu et al [4] demonstrated the feasibility and utility of monitoring the expression of miRNAs in human cancer tissue. They found a high level of diversity in miRNA expression across cancers, and found that approximately 200 miRNAs could be sufficient to classify human cancers. Tam [5] adds that because miRNAs function as managers in gene regulatory networks, they are distinct from other biomarkers because they have a pathogenic role in the disease process and are not by-products of the disease state. Because miRNAs function by specific binding to their targets, polymorphisms (SNP) within the sequence of miRNAs or their target mRNAs can lead to disease, including cancer. These miRNA-specific SNPs can influence the risk of disease and can also be used in the diagnosis of these diseases [6]. Deregulated miRNAs have been described from numerous human cancers including breast, lung, colon, ovarian, and prostate cancer. Mitchell et al [7] found that miRNAs originating from prostate cancer tissue enter circulation and can be used to distinguish patients with prostate cancer from healthy controls and established a blood-based PCR approach for the detection of human prostate cancer. A similar approach was used to detect serum miRNA from ovarian cancer patients [8]. Taylor and Gercel-Taylor [9] investigated the use of circulating exosomes in the diagnosis of cancer. They found that the miRNA content of ovarian tumor cells and circulating exosome was similar and could be used to distinguish cancer patients from patients with benign ovarian disease and from normal controls.

miRNA signatures from normal and cancerous tissues have been used to classify several types of cancer and may also allow clinicians to determine a treatment course based on the original tissue type. It may also be possible to use miRNA expression patterns as a biomarker to monitor the effect of therapy on cancer progression [2]. Because miRNA expression profiles parallel the developmental origins of tissues, and because relatively few miRNAs can be used to effectively type tissues, they are potentially superior markers than messenger RNAs for cancer diagnosis [4]. The potential for the use of serum miRNAs as biomarkers of disease and as targets of therapeutics is promising [10], since it would mean a non-invasive, accurate test for cancer. In this study, we demonstrate that serum miRNAs can be used to discriminate between cancer patient sera and normal donor sera, using a simple microarray assay that requires no amplification step.

## Results

### Serum microRNA extraction

We have determined that a sufficient quantity of miRNAs is present in less than 1 ml of human serum to produce a detectable signal on a microarray using fluorescence or electrochemical detection (ECD). Using a simple phenol/chloroform extraction protocol, we recovered approximately 1.3 µg of serum RNA from each 800 µl of serum (average of 18 samples; standard deviation 0.3). The resulting pattern of miRNA expression could be used to distinguish between cancer patients and normal donors.

The approximate size of the small RNAs recovered from plasma was determined by isolating large RNA fragments (low ethanol concentration) and small RNA fragments (high ethanol concentration) using the Invitrogen PureLink miRNA isolation kit, after acid phenol/chloroform extraction and precipitation. The two RNA size fractionations were labeled with biotin (Mirus) and hybridized to a microarray. Results (not shown) indicated that the vast majority of signal was from the small RNA fraction, which was similar to the signal from the un-fractionated sample.

DNA contamination of extracted serum nucleic acid was examined by comparison of an extracted sample that was split, and half treated with DNase I. After labeling both treated and untreated samples with biotin and hybridizing to different sectors of the same 4×2K array, very little difference could be seen between treated and untreated samples (r^2^ = 0.9 and 0.96 respectively for two replicates) indicating that little DNA contaminates samples (data not shown).

### Assay sensitivity and stability

**Figure 1:**
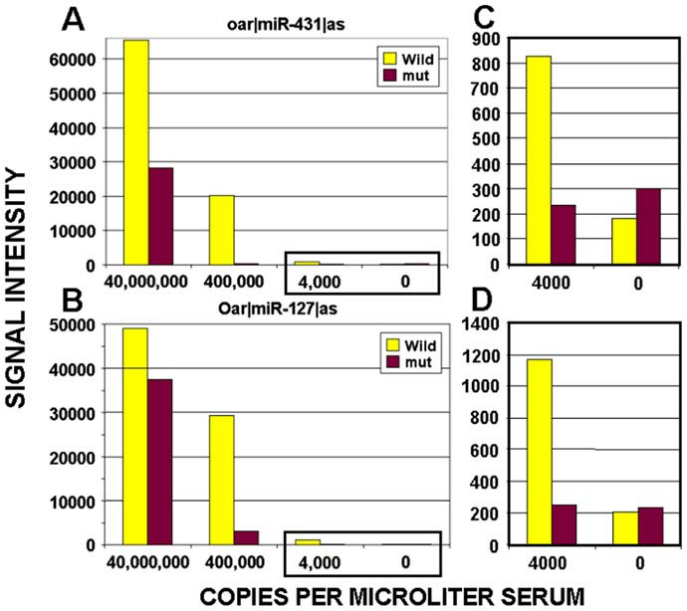
Assay sensitivity. RNA miRNA analog oligonucleotides, at concentrations ranging from 0 to 40 million copies per microliter, were spiked into 400 ul of serum after the addition of RLT buffer. RNA was then extracted from the serum using phenol/chloroform extractions and an ethanol precipitation. Samples were then labeled and hybridized on a microarray. Vertical bars indicate array signal intensities for specific miRNA probes representing the wild type sequence (Wild) and probes with two internal mutations (mut) for (A) oar|miR-431 and (B) oar|miR-127. Scales for the 4,000 and 0 copies data points (boxed in left panels) are expanded in the right panels: (C) oar|miR-431, and (D) oar|miR-127.

The sensitivity of our miRNA assay was determined by adding dilutions of a synthetic RNA oligonucleotide to our assay during serum extraction. We were able to detect approximately 4,000 copies of serum microRNAs per microliter of serum (Figure 1). This detection level is similar to that reported in Mitchell et al [7] for the prostate cancer specific microRNA miR-141 using TaqMan assays. miRNA microarrays are relatively more sensitive than standard expression microarrays because small oligonucleotides tend to have better hybridization kinetics than larger RNA or DNA molecules. For miRNAs, both their protection from digestion by various cellular factors, and their small size contribute to their detection in serum by microarrays at levels that are as low as those seen with methods that would otherwise be considered more sensitive.

We have also determined that data collected from the same serum samples after being frozen at −80°C for 1 week after the initial microRNA assays, was similar to the original data. MicroRNAs from aliquots of 2 serum samples from cancer patients (1 prostate and 1 colon) were extracted, labeled and hybridized to arrays, and after one freeze/thaw event, new aliquots were again extracted, labeled and hybridized to a second array. Data sets, from re-assayed prostate cancer sample 811 and colon sample 792, showed strong correlations when raw array data were compared (r^2^ = 0.94 and 0.96 respectively). This result indicates that the assay is reproducible and stable over time.

### Serum microRNA data analysis

**Figure 2:**
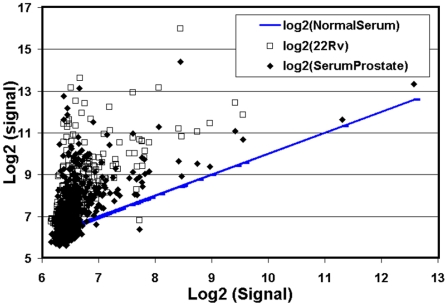
Up-regulation of cancer sera miRNAs over normal donor sera miRNAs. Log transformed normal donor serum miRNA signals (blue line) were compared to miRNA array signals from a prostate cancer cell line 22Rv (open squares) and from a prostate cancer patient (closed diamonds). In general, cancer and cell line miRNAs seem to be up-regulated when compared to normal donor serum miRNAs.

Several prostate, ovarian, colon, breast and lung cancer serum samples as well as normal male and female donor sera have been analyzed on the pan-miRNA microarray to ascertain serum miRNA profiles and to confirm the specificity of the profiles for different cancers and normal donors. Several data analysis methods have been tested to determine the most relevant method for the discrimination of cancer versus normal. For preliminary analysis, we log_2_-transformed serum miRNA probe signals from a normal donor and compared this data to log_2_-transformed probe data from a prostate cancer patient and from a prostate cancer cell line. Although both the prostate cancer serum sample and the prostate cancer cell line (22Rv1) sample showed up-regulation compared to a normal serum sample, they did not show much similarity to each other. At this point, we simply note a relative up-regulation of serum miRNAs in cancer as compared to serum from normal donors (Figure 2).

**Figure 3:**
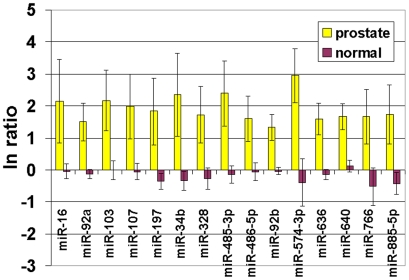
Analysis of microRNA data for normal and prostate cancer sera. After data set normalization, the natural log of the ratio of the signal for a specific probe over the same probe from the normal serum sample was taken. 15 miRNAs showed up-regulation in all stage 3 and 4 prostate cancer samples when compared to sera from normal male donors. These miRNAs are listed below each data set. Five stage 3 and 4 prostate cancer sera (Yellow), and 8 normal male donor sera (red) were analyzed. Vertical lines indicate plus or minus one standard deviation of the mean.

We first set out to define a minimal set of probes that would allow us to discriminate between prostate and normal serum samples. Signal from each miRNA probe was first background corrected using negative control probes. Subsequently, each miRNA probe was expressed as the natural log of the ratio between itself and the same probe in a normal human male serum sample. This gives a value of 0 for all the base serum sample probes and an up or down regulation with respect to that sample (normal) for all the other samples (normal and cancer). Figure 3 shows the ratios of a subset of probes that passed multiple criteria. All prostate cancer probe data sets were filtered to remove all probes whose perfect match signal was not greater than its mutant signal. Fifteen miRNAs were found to be over-expressed in serum from all stage 3 and 4 prostate cancer patients (miR-16, -92a, -103, -107, -197, -34b, -328, -485-3p, -486-5p, -92b, -574-3p, -636, -640, -766, -885-5p) with respect to 8 normal controls (Figure 3). This analysis also showed a slightly elevated signal for miR-141 in stage 3 and 4 prostate cancer patient sera (mean = 829, STD = 201) over normal donor sera (mean = 555, STD = 64); which is in agreement with data reported by Mitchell et al [7] using RT-PCR.

**Figure 4:**
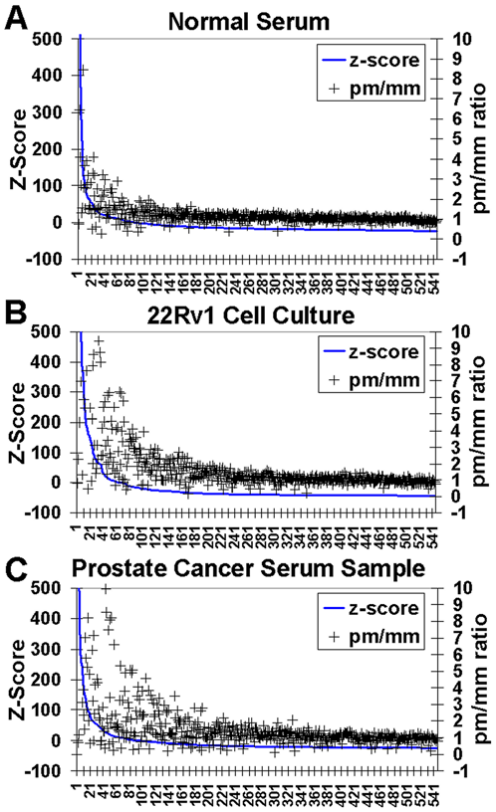
Analysis of signal from perfect match (wild type) and miss-match (double mutant) miRNA probes (pm/mm ratio). (A) Analysis of signal from normal serum; (B) Analysis of signal from 22Rv1 cell culture; and (C) signal from prostate cancer patient serum. Z-scores (blue lines) were determined by subtracting the signal at each probe by the mean of the test probes from the entire hybridization, and then, by dividing the resulting value by the standard deviation of the signal across test probes, across the entire hybridization.

In Figure 4, we have plotted z-score-corrected signal intensities for three hybridizations. Z-score normalization was computed by subtracting the signal at each probe by the mean of the test probes from the entire hybridization, and then, by dividing that value by the standard deviation of the signal across test probes, across each hybridization. This normalization yields probe signals that are centered and normalized to a mean of 0 and a standard deviation of 1.0. For each plot, signal was sorted from highest to lowest intensity, and plotted as a solid line. Perfect match/mismatch (pm/mm) ratios were plotted as open triangles over the signal intensity line. Clearly, probes with higher intensities have significantly higher pm/mm ratios. For signal from a miRNA to be considered significant, its perfect match (wild-type anti-sense) probe signal must be greater than that of its double mutant negative control (mm) probe. Specifically, the pm/mm ratio must be greater than 1.5 (plotted on the right-hand side y-axis). By this metric, at least 34 probes were considered significant for the normal serum sample (Figure 4A). For the prostate cancer cell line 22Rv1 sample and prostate cancer serum sample (Figure 4 B and C), there were 57 and 62 significant probes respectively. At the simplest level, the fact that most of the probes that have high signal also have high pm/mm ratios, indicates that the signals we are reading are real. We also performed several non-miRNA negative control hybridizations. For these hybridizations, although some probes have a higher signal than others, this is not accompanied by a corresponding increase in the pm/mm ratios (not shown).

#### Hierarchical clustering

**Figure 5:**
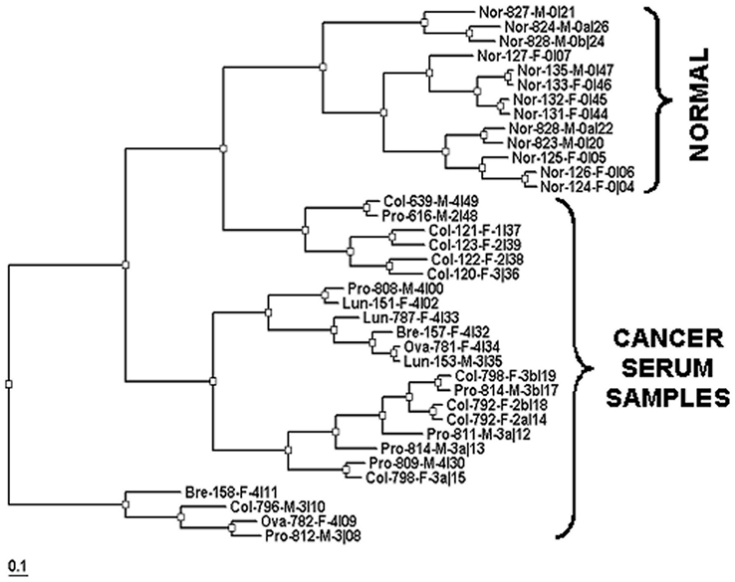
Hierarchical clustering of microarray data (Spearman). Cancer samples and normal donor samples (brackets) were clustered using a hierarchical clustering program to show sample-to-sample relationships. Sample labels include donor condition (cancer type or normal), sample lot number (last three digits), gender, and cancer stage (2 – 4, or 0 for normal). Labels marked with a or b indicate repeat testing of the same sample.

Hierarchical clustering was used to group samples from different disease and normal states (Figure 5). The dataset used for clustering contained 35 serum samples that were a mixture of normal serum and cancer serum samples of diverse types and severity (stages) (see Tables 1 and 2). Since most of the miRNAs used on the microarray are not present at detectable levels in most serum samples, clustering was only performed on a subset of the miRNAs. This subset was drawn from the group of miRNAs that were judged to be significant. Only signal from miRNA probe sets that were found to have been significant in at least 5 hybridizations across the entire data set were taken and used for further analysis. Signal from test probes (wild-type anti-sense) was log_2_-transformed. These probes were then normalized by conversion to Z-score. Only test probes, not any of the negative controls or spike-ins, were used for this calculation. Signal was then thresholded based on significance. Hierarchical clustering was performed using a program written in-house that uses the spearman rank correlation as the distance function. The output for this program is a dendrogram that was displayed using the program Treeview [11]. Clustering indicates a clear demarcation between normal and most cancer samples (Figure 5).

**Table 1:** Summary of human cancer serum samples tested in this study.

CANCER PATIENT SERUM SAMPLES					
LOT NUMBER	GENDER	AGE	STAGE	CANCER TYPE	TREATMENT
BRH233781	Female	63	4	OVARIAN CANCER	Carboplatin, Taxotere
BRH233782	Female	66	4	OVARIAN CANCER	Carboplatin, Gemzar
BRH233787	Female	69	4	NON-SMALL CELL LUNG	Zofr, Deca, Carb, Neul, Veps
BRH234151	Female	68	4	SMALL CELL LUNG	Topotecan
BRH234153	Male	62	3	SMALL CELL LUNG	Zometa
BRH237121	Female	69	1	COLON CANCER	None
BRH237122	Female	67	2	COLON CANCER	Ferrlecit
BRH237123	Female	85	2	COLON CANCER	None
BRH233792	Female	63	2	COLON CANCER	None
BRH237120	Female	87	3	COLON CANCER	Zometa
BRH233796	Male	69	3	COLON CANCER	5FU
BRH233798	Female	76	3	COLON CANCER	None, Pretreatment
BRH249639	Male	47	4	COLON CANCER	5FU
BRH234157	Female	80	4	BREAST CANCER	Femara, Zometa
BRH234158	Female	44	4	BREAST CANCER	Xeloda, Zometa
BRH233808	Male	65	4	PROSTATE	Taxotere, Zometa
BRH233809	Male	62	4	PROSTATE	Taxotere, Zometa
BRH233811	Male	72	3	PROSTATE	Lupron, Zometa
BRH233812	Male	61	3	PROSTATE	None
BRH233814	Male	59	3	PROSTATE	Taxotere
BRH249616	Male	64	2	PROSTATE	None

**Table 2:** Summary of normal donor serum samples tested in this study.

NORMAL DONORS		
LOT NUMBER	GENDER	AGE
BRH233823	Male	40
BRH233824	Male	61
BRH233825	Male	44
BRH233826	Male	42
BRH233827	Male	49
BRH233828	Male	50
BRH233829	Male	41
BRH237135	Male	31
BRH237124	Female	56
BRH237125	Female	22
BRH237126	Female	22
BRH237127	Female	69
BRH237131	Female	48
BRH237132	Female	47
BRH237133	Female	32

#### Heat Map Analysis

**Figure 6:**
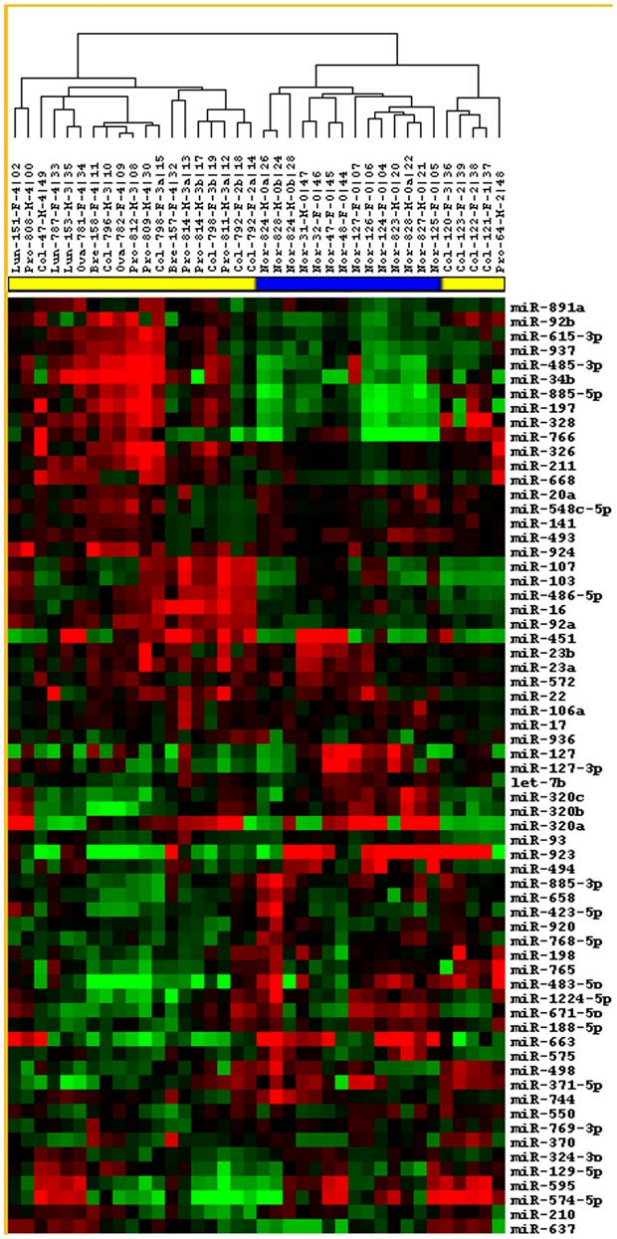
Heat maps of microRNA array data. The set of miRNAs used for analysis was chosen based on significance in at least 5 hybridizations. A probe-set was judged significant if the ratio of perfect-match (PM)/mismatch (MM) probes was greater than 1.5.For each hybridization in the analysis, only significant signal was used for the clustering. Signal for those miRNAs whose signal was judged significant by PM/MM ratios was Log 2 converted. Then the signal was median normalized over that hybridization and Average Linkage Clustering was performed using a Spearman Rank Correlation. Clustering was visualized using the program TreeView [12]. Green indicates negative values and Red indicates positive values. Samples are identified as either normal (blue bar) or cancer (yellow bar) at the top of the figure. miRNA names are listed to the right and sample identifications are listed above.

To further explore the miRNAs responsible for the clustering, Heat maps were used to look for similarities between miRNA expression patterns within each sample. This method is most effective when rows and columns are ordered to allow these patterns to be easily identified. Clustering was thus used to give this ordering (by identifying miRNAs that have similar expression patterns, and arranging them in close proximity). This data was ported to the open source program, Cluster [12]. The raw data for both sample and miRNA signal were median centered and then clustered using average linkage, spearman rank coefficient as a distance function. Heatmaps were displayed using Treeview [11]. This method resulted in a clear ordering of the samples taken from our test-set (Figure 6). Samples were labeled with a unique identifier. Serum samples from patients with colon, prostate, ovarian, breast and lung cancer in various stages of disease and treatment were used in this dataset. This analysis resulted in two main branches: one major cluster of sequences containing most of the cancer samples, and a second branch containing the normal group along with a second cancer group (Figure 6).

#### Decision Tree Analysis

For each miRNA mature region, two controls were written on the chip: a sense wild-type probe (s), an anti-sense wild-type (PM) form and a double mutant control (MM). This probe set was used to evaluate significance as well as intensity of each miRNA mature form. For each hybridization, the raw signal was extracted and probes were grouped by the miRNA that they were designed for. PM signal was log_2_ transformed, and Z-normalized. For the classifier and for the focused cancer versus normal hierarchical clustering, we used a simple heuristic to determine if a probe was indeed present. For each miRNA that was evaluated, we counted a signal significant if the signal for PM/MM >1.0. If so, then the Z-normalized log_2_ (signal) was used for that miRNA, otherwise the probe data for that particular probe was not used. Normalization was thus performed over anti-sense wild-type probes across the chip; however, only data from significant probes were used for clustering and classification.

Data Mining was performed using the WEKA package [13]. Normalized probe intensity data was converted into ARFF format and input into the WEKA program. Hybridizations were grouped into two classes, ‘cancer’ and ‘normal’. We used the CfsSubsetEval routine to choose miRNA's (attributes). This method gave the best classification of the data into the two classes. This routine assesses the predictive ability of each attribute as well as the degree of redundancy among each miRNA. It prefers attributes that are highly correlated within each class, but that have low inter-correlation. The choice of attributes was performed using 10-fold cross-validation, and selection of the 28 best attributes was based upon their being selected at least 30% of the time.

**Figure 7:**
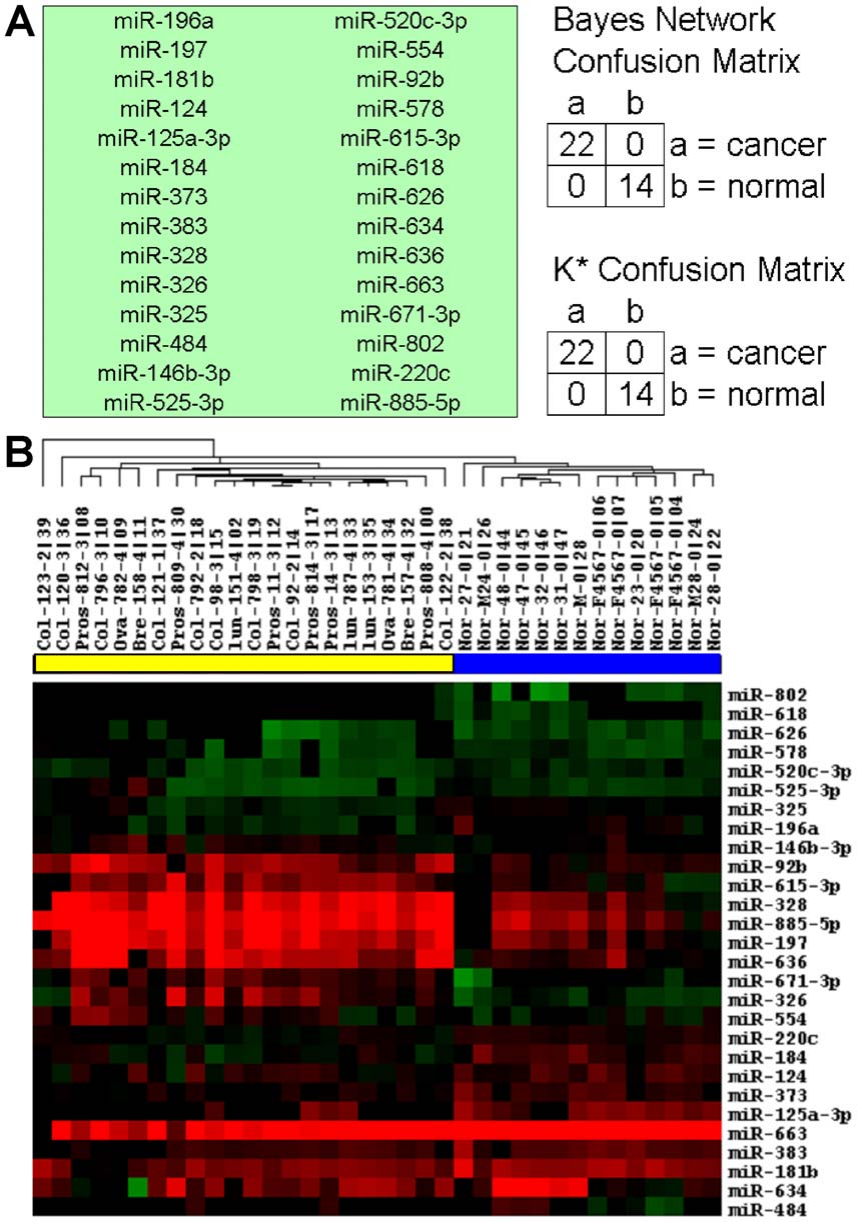
Cancer vs. Normal Classifier. Data Mining was performed using the WEKA package [13]. miRNA probes (attributes) were chosen using an attribute selection routine called CfsSubsteEval. (A) 28 miRNAs were found using this method. Two distinct classifier methods, both the Bayes network implementation and K* methods [14] were able to correctly classify these samples (using 10-fold cross-validation). The results for both classifier runs are displayed as a confusion matrix on the right. (B) Hierarchical clustering of normal and cancer samples was performed using signal from these 28 miRNAs. Red blocks are highly expressed, green are considered down-regulated and black blocks are non-significant as judged by PM/MM criteria described in the text. Samples are identified as either normal (blue bar) or cancer (yellow bar) at the top of the figure. miRNA names are listed to the right and sample identifications are listed above.

We next extracted signal from each hybridization for just this selected set of miRNA's. In Figure 7A, two different classifiers were used to distinguish cancer vs. normal samples. A Bayesian network and an instance-based method called K* [14] were able to clearly differentiate the two classes within 36 samples. Clustering was next performed using the Cluster program from Eisen et al. [12]. Average linkage was used as the tree-building criteria and Spearman rank correlation was used as the distance function. Figure 7 B shows a much-improved separation of normal vs. cancer patient samples.

#### Analysis of coded samples

miRNA microarray data from cancer patients and normal donor sera were coded to remove any indication of disease status and submitted for analysis. Using the subset of attributes described above and in Figure 7, 16 single-blinded samples were added to the dataset and analyzed with the classifier. The k-star classifier was able to call 16/16 samples correctly as either from cancer patients or normal donors, while the Bayes Network classifier produced 3 misclassifications out of 16 (data not shown).

## Discussion

miRNA expression signatures have a potential role in the diagnosis, prognosis and therapy of human diseases, including cancer, heart disease, viral infections and inflammatory diseases [1]–[4], [10], [15]–[25]. Deregulation of miRNAs in cancer can be caused by chromosomal deletions, amplifications and translocations; by hypermethylation of CpG islands; and by regulation of transcription and post-transcriptional processing [1], [2], [23]. Aberrant expression of miRNAs can influence cancer progression by affecting the expression of oncogenes or tumor suppressors, and miRNAs, such as the miR-17-92 cluster, can function directly as oncogenes [1], [2], [23]. miRNAs are also involved in cancer through their effect on the cell cycle, apoptosis, metastasis, and angiogenesis [1], [2], [23].

Several studies have detailed the miRNAs that are associated with cancers [15], [23], [26]–[32]. Most of these studies have used biopsy samples, archival tissues or cancer cells [5], [33]–[37]; or have used miRNAs extracted from paraffin embedded or formalin fixed tissues [38]. By comparing non-cancerous tissues surrounding cancerous tissues, or normal donors versus cancer patients, those miRNAs that are up or down regulated can be identified, often after PCR amplification. Many studies have been published describing the specificity of miRNAs for different types of cancers, cancer stages and cancer treatments and several reviews have been published that summarize the most recent information on the roles of miRNAs in cancer [1], [2], [5], [6], [15], [23], [25], [32]. These studies demonstrate the utility of miRNAs in both diagnosis and prognosis of several cancers and also differentiate between cancer and benign disorders [34]. While numerous studies have led to an understanding of miRNAs at a tissue or cell level, there is a paucity of data in serum studies hampering their use in routine diagnosis.

Recently a series of studies has been performed on miRNAs present in serum [7]–[9], [39]–[41]. In a recent report, miRNAs were shown to be significantly elevated in pregnant versus non- pregnant women [40]; and in another study, placental miRNAs were shown to be present in maternal plasma [42]. In these studies, the miRNAs were found to be very stable to storage, and also to any RNAse degradation. miRNAs have recently been shown by Mitchell et al [7] to circulate in serum of prostate cancer patients [7]. In particular, miR-141 could differentiate prostate cancer patients from normal individuals [7]. In a work by Taylor and Gercel-Taylor [9], circulating tumor exosomes were isolated from serum of ovarian cancer patients using magnetic beads and an antiEpCAM antibody. miRNAs were then extracted, labeled and detected by microarray. This approach, using larger volumes of serum, indicated that eight diagnostic miRNAs were up-regulated in cancer exosomes: miR-21, miR-141, miR-200a, miR-200c, miR-200b, miR-203, miR-205, and miR-214. To date however, no routine assay is available for examining miRNA signatures in serum or in the plasma of cancer patients.

One issue that appears in these studies is the fact that the miRNA expression patterns seen in serum are not identical to those seen from miRNAs taken directly from cancer cell lines. In our study, we found that although both the prostate cancer serum sample and the prostate cancer cell line (22Rv1) sample showed up-regulation compared to normal serum sample, they did not show much similarity to each other. This seeming discrepancy could be taking place for a number of reasons. The most obvious of which is the possibility that samples taken from the cell lines themselves are not representative of what appears in the serum. We can speculate that the most obvious source of miRNAs that appear in the serum is a product of tumor cell lysis; however, it may also be possible that their appearance in the serum is the product of a form of active transport involving the formation of exosomes (36). This would confound a direct comparison between miRNA expression patters derived from cell-line and tumor with those that are serum-derived.

Information on the use of miRNAs as biomarkers is predominantly associated with studies on tissue samples or cancer cell lines. Distinct patterns of miRNA expression are able to distinguish between cell type and stage in various cancers. This bodes well for diagnostic and prognostic applications of miRNA profiles. It also indicates there is clearly a need to define the expression profiles of miRNAs in serum of cancer patients and compare these to profiles observed in the serum of individuals representing a range of diseased and healthy states. It is anticipated that miRNA profiles in serum have the potential to be early markers for cancer detection and will also play a role in the monitoring of disease status during chemotherapy.

In this study, we have determined that a sufficient quantity of miRNAs is present in one ml of human serum to produce a detectable signal on a microarray using fluorescence or electrochemical detection. At the simplest level, this study has shown that serum miRNAs are up-regulated in cancer patients as compared to normal donors. In a comparison of stages 3 and 4 prostate cancer sera and normal donor serum miRNA levels, we found that 15 miRNAs (miR-16, -92a, -103, -107, -197, -34b, -328, -485-3p, -486-5p, -92b, -574-3p, -636, -640, -766, -885-5p) were up-regulated in serum from prostate cancer patients compared to normal donor sera.

Heat Map and cluster analyses show that serum miRNA signatures can also be used to separate cancer patients and normal donors in most cases. Sixty-five miRNAs (see Figure 6) were used in a Heat Map analysis that isolated 21 cancer samples, plus 3 re-assayed samples, from normal samples. Sixteen cancer samples clustered together, while five cancer samples formed a separate cluster within the normal sample cluster. Cluster analysis (see Figure 5) was also used to distinguish 21 cancer samples from 12 normal samples. Three cancer samples that were re-analyzed after freeze-thawing and storage, also clustered with the cancer samples

We have also shown that serum miRNAs can be detected at a level similar to that reported for TaqMan PCR from serum, approximately 4,000 copies per ul [7], and that the assay is stable for repeated sample testing after storage. The relative sensitivity of miRNA microarrays over standard expression arrays could be due to the hybridization kinetics of small oligonucleotides. Small molecules tend to have better hybridization kinetics than larger RNA or DNA molecules. Indeed, most of the thermodynamic models for predicting DNA hybridization behavior were developed using short oligonucleotides [43]. Many of the conclusions drawn from such studies do not apply when larger molecules are used as secondary structure, non-specific binding, and various other unforeseeable effects interfere with predicted hybridization behavior. For miRNAs, both their protection from digestion by various cellular factors, and their small size contribute to their detection in serum by microarrays at levels that are as low as those seen with methods that would otherwise be considered more sensitive, such as RT-PCR. The sensitivity of the microarray platform used here has allowed us to monitor miRNA from a small volume of serum and to classify sera as either from normal donors or from cancer patients.

Our results, in general, agree in many cases with previously published studies. However, differences in our results from other studies could result from several factors: 1) Serum miRNA expression profiles do not directly correspond to tissue profiles. The low levels of miRNAs in serum are better suited to studies of up-regulation and not down-regulation. In addition, it is unlikely that there is a direct correspondence between tissue miRNA levels and serum miRNA levels due to the possible mechanisms of miRNA release into circulation (cell lysis or exosome release [9]) [8], and to the release of miRNAs into circulation from other tissues as a result of the cancer or other related or non-cancer related conditions; 2) Different miRNA labeling techniques can produce different expression profiles. For example, ULS chemical labeling (Mirus and Kreatech kits), which only targets G residues, produces signal intensities that are directly proportional to the number of G residues in the miRNA. In contrast, labeling by incorporating modified nucleotides or label into an extended poly A tail, or by labeling with a modified primer by first strand cDNA synthesis, should produce a more even label; 3) We found that amplification techniques such as RT-PCR or T7 promoter expression, produce different expression patterns on arrays when compared to un-amplified samples. This could be due to differential efficiency in the amplification process. In addition, the limited quantity of miRNAs in human serum is not sufficient for column purification, which is commonly used for tissue miRNA purification.

An example of published data from two different miRNA expression profiling techniques that do not show strong agreement is illustrated in the following comparison. Schetter et al [44] labeled colon cancer tissue miRNAs by reverse transcriptase extension with a labeled primer and hybridized the target to a microarray, while Monzo et al [45] determined colon cancer tissue miRNA expression levels by TaqMan RT-PCR. When the 26 up-regulated miRNAs from the Schetter study are compared to those from the Monzo study, only 14 miRNAs are in agreement (54%). In a similar comparison of up-regulated prostate cancer tissue miRNAs from studies by Tong et al (TaqMan data [46]), Porkka et al (array data [47]), and Ambs et al (array and TaqMan data [33]), very little overlap of miRNA expression data can be seen: little or no overlap in data between Tong et al and Porkka et al, and only 1 of 33 and 1 of 34 miRNAs were in agreement for Tong et al compared to Ambs et al, and for Porkka et al compared to Ambs et al, respectively. Of the 15 upregulated serum miRNAs we report for prostate cancer, 4 are in agreement with data from Ambs et al, and 2 are in agreement with data from Porkka et al.

This study must be confirmed with a larger and better-documented data set. We do not yet know the affects of gender, age and cancer treatment on miRNA levels in serum. Radiation and chemotherapies that result in remission of cancer should also result in a change in the serum miRNA profiles. Wong, et al. [48] have shown that plasma levels of miR-184 were elevated in patients with squamous cell carcinoma of the tongue, and that plasma miRNA levels were reduced after surgical removal of the tumor. This would indicate that cancer treatment does have an affect on the levels of cancer-specific miRNAs in circulation. The samples used in this study were from cancer patients that were treated by chemotherapy in most cases. Because we do not have detailed information on the results of treatment on cancer progression or remission we cannot include this variable in our analysis. Additional studies with well-documented patient samples will be needed to address this question.

## Materials and Methods

### DNA array synthesis and microarray configuration

**Figure 8:**
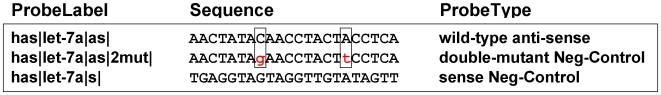
Probe design scheme for miRNA array. Sequences for miRNA probes were taken from the Sanger database version 10.0 (released, 8/2/2007). We generally used only the predominantly expressed form for each miRNA precursor. This was done to save space on the array. The final list included all of the dominant miRNA forms from the studies referenced in this paper [4], [8], [18]. Three probes were designed for each mature miRNA sequence: 1) antisense to wild type; 2) double mutated antisense (boxes); and 3) sense negative control probes (included if the corresponding sequence was not found in miRNA databases). Hsa-let-7a is used here as an example of our approach to probe design.

Arrays for serum miRNA analysis were constructed with 547 human miRNA sequences obtained from the Sanger Database version 10.0 which appeared on 8/2/07. Because of limited space on the array, the probe list was modified to exclude a few newer miRNAs that had recently been added. The array includes miRNA probes for all studies referenced by this paper. Three probes were written for each miRNA: an anti-sensed wild-type version, a double-mutant control probe, and a sense control version (Figure 8). Antisense controls were not included if the corresponding antisense miRNA existed in the databases. The double mutant control mutations were screened in order to maintain the same notional melting temperature (Tm) as the wild-type Tm. They were also designed to avoid perturbing or creating any secondary structure that might appear in the wild-type probe. In addition to the 547 human miRNAs, we also included as controls, four sheep, three *C. elegans*, and two human sequences. These arrays have been initially evaluated using a Cy5 fluorescence detection system, but can be converted to a more sensitive electrochemical system (ECD: ElectraSense^®^) [49]. Additional microRNAs can be easily added to the array as they are identified in the Sanger database.

### 22Rv1 cell culture

22Rv1 human prostate cancer-derived cells were cultured in standard plastic tissue culture plates in RPMI medium 1640 (GIBCO) supplemented with 10% FBS and 1% penicillin-streptomycin at 37°C in a 5% CO_2_ incubator. Cells were harvested in Qiagen RLT buffer and extracted with phenol/chloroform as described below.

### Human serum samples

Human serum samples were purchased from Bioreclamation, Inc, Hicksville, NY and include: stages 2 to 4 prostate, stages 1 to 4 colon, stage 4 ovarian, stage 4 breast, and stages 3 and 4 lung cancer sera (Table 1); and sera from normal male and female donors (Table 2). All cancer samples have associated patient data including age, race, gender, chemotherapy, and stage of disease. No information on treatment outcome or on radiation therapy was supplied with samples. Samples were stored at minus 80°C until use.

### MicroRNA extraction, labeling and hybridization

#### Extraction

An aliquot of 400 µl of each serum sample was mixed with 500 µl lysis buffer (RLT, Qiagen, Valencia, CA) and 800 µl acid phenol: Chloroform (Ambion, Foster City, CA), vortexed for 30 seconds and centrifuged at 16000 rcf for 10 min at 25°C. The aqueous phase was extracted 2× with an equal volume of acid phenol:chloroform and centrifuged at 16000 rcf for 10 min at 25°C. The resulting aqueous phase was then precipitated with 0.1 vol 5 M NaCl, 2 µl precipitation enhancer (Mirus, Madison, WI), 2 µl GlycoBlue (Ambion) and 2.5 vol 100% ethanol at −20°C for at least 1 hr. After centrifugation at 4°C for 30 min, the pellets were washed 2× with 75% ethanol and then air-dried. Precipitated RNA was resuspended in 50 µl molecular grade water (Ambion) and quantified with a NanoDrop ND-1000 spectrophotometer (Thermo Scientific, Wilmington, DE).

#### Labeling

Approximately one µg of isolated RNA was labeled with a Mirus miRNA Biotin labeling kit (MIR8450) following manufacturers directions. Briefly, 1 µg RNA was diluted to 86 µl with water and 10 µl of 10**×** buffer was added followed by 4 µl LabelIT biotin labeling reagent and incubation at 37°C for 1 hr. The reaction was stopped with 10 µl stop reagent and the sample precipitated as described above. The dried pellet was resuspended in 5.1 µl water.

#### Hybridization

Sectored array chambers (4 chambers per array) (CombiMatrix 4×2K arrays™) were each filled with 30 µl of Pre-Hybridization Solution (CombiMatrix Corp) and incubated for 10 min at 45°C. MicroRNA was mixed with 9 µl 20× SSPE (Ambion), 4.8 µl BSA at 50 mg/ml (Ambion), 3.6 µl deionized formamide (Sigma) and 7.5 µl of 10% SDS (Ambion) and heated to 95°C for 3 min. 30 µl of each sample were added to sectored hybridization chambers, sealed with aluminum tape, and incubated at 45°C for 16 hr with rotation. After hybridization, arrays were washed 2× with 2× SSC with 0.1% SDS at room temperature (RT) for 10 sec (CombiMatrix Corp), 2× with 2× SSC at RT for 10 sec and then washed 1× with 0.2× SSC at RT for 10 sec each.

Arrays were blocked with 5× PBS/Casein Blocking Buffer at RT for 10 min and then labeled with either Cy5 labeling solution for fluorescence scanning or HRP Biotin Labeling Solution (CombiMatrix) for ElectraSense reading (CombiMatrix) and incubated for 30 min at RT. Arrays were then washed 2× with Biotin Wash Solution (2× PBST) for 30 sec each at room temp and again washed 2× with 2× PBS followed by scanning for fluorescence, or washed 2× with TMB Rinse Solution (CombiMatrix), followed by one wash with TMB substrate (CombiMatrix) and scanning with an ElectraSense reader (CombiMatrix) after fresh TMB was added.

### Determination of assay sensitivity

To determine the sensitivity of our assay, commercially purchased RNA miRNA analog oligonucleotides (IDT), at concentrations ranging from 0 to 40,000,000 copies per microliter, were spiked into 400 µl of normal human serum after the addition of RLT buffer. RNA was then extracted from the serum using acid phenol/chloroform extractions and an ethanol precipitation. Samples were then labeled with biotin and hybridized on a microarray as previously described.

The approximate size of the small RNAs recovered from serum was determined by isolating large RNA fragments (low ethanol concentration) and small RNA fragments (high ethanol concentration) using the Invitrogen PureLink miRNA isolation kit, after phenol/chloroform extraction and precipitation. The two RNA size fractionations were labeled with biotin (Mirus) and hybridized to a microarray as described above.

### DNA contamination of extracted serum nucleic acids

Purified nucleic acid from a serum sample was split into two aliquots (DNase I-treated and untreated). One aliquot was digested with DNase I (New England Biolabs, Ipswich, MA) for 30 min at 37°C, following manufacturer's protocol, and then heated to 85°C for 15 min. This sample was then precipitated with NaCl and ethanol and both DNase I-treated and untreated samples were labeled with a Mirus biotin-labeling kit as described above.

### GEO Database

Array data accession numbers: GPL8686; GSE16512; GSM414832 - GSM414867.

## References

[1] Nelson KM, Weiss GJ (2008). MicroRNAs and cancer: past, present, and potential future.. Mol Cancer Ther.

[2] Lee YS, Dutta A (2009). MicroRNAs in Cancer.. Annu Rev Pathol Mech Dis.

[3] Asli NS, Pitulescu ME, Kessel M (2008). MicroRNAs in organogenesis and disease.. Curr Mol Med.

[4] Lu J, Getz G, Miska EA, Alvarez-Saavedra E, Lamb J (2005). MicroRNA expression profiles classify human cancers.. Nature.

[5] Tam W (2008). The Emergent role of MicroRNAs in molecular diagnostics of cancer.. J Molec Diag.

[6] Chen K, Song F, Calin GA, Wei Q, Hao X, Zhang W (2008). Polymorphisms in microRNA targets: a gold mine of molecular epidemiology.. Carcinogenesis.

[7] Mitchell PS, Parkin RK, Kroh EM, Fritz BR, Wyman SK (2008). Circulating microRNAs as stable blood based markers for cancer detection.. Proc Nat Acad Sci USA.

[8] Resnick KE, Alder H, Hagan JP, Richardson DL, Croce CM, Cohn DE (2009). The detection of differentially expressed microRNAs from the serum of ovarian cancer patients using a novel real-time PCR platform.. Gynecol Oncol.

[9] Taylor DD, Gercel-Taylor C (2008). MicroRNA signatures of tumor derived exosomes as diagnostic markers of ovarian cancer.. Gynaecologic Oncology.

[10] Jackson DB (2009). Serum-based microRNAs: Are we blinded by potential?. PNAS.

[11] Page RDM (1996). TREEVIEW: An application to display phylogenetic trees on personal computers.. Comput Appl Biosci.

[12] Eisen MB, Spellman PT, Brown PO, Botstein D (1998). Cluster Analysis and Display of Genome-Wide Expression Patterns.. Proc Natl Acad Sci U S A.

[13] Witten IH, Frank E (2005).

[14] Cleary JG, Trigg LE (1995).

[15] Chin LJ, Slack FJ (2008). A truth serum for cancer- microRNAs have major potential as cancer biomarkers.. Cell Research.

[16] Corney DC, Nikitin AY (2008). MicroRNA and ovarian cancer.. Histol Histopathol.

[17] Dahiya N, Sherman-Baust CA, Wang T-L, Davidson B, Shih L-M (2008). MicroRNA expression and identification of putative miRNA targets in ovarian cancer.. PLos one.

[18] Rosenfeld N, Aharonov R, Meiri E, Rosenwald S, Spector Y (2008). MicroRNAs accurately identify cancer tissue origin.. Nature Biotechnology.

[19] Houzet L, Yeung ML, de Lame V, Desai D, Smith SM, Jeang KT (2008). MicroRNA profile changes in human immunodeficiency virus type 1 (HIV-1) seropositive individuals.. Retrovirology.

[20] Sheedy FJ, O'Neill LA (2008). Adding fuel to fire: microRNAs as a new class of mediators of inflammation.. Ann Rheum Dis.

[21] Bi Y, Liu G, Yang R (2009). MicroRNAs: novel regulators during the immune response.. J Cell Physiol.

[22] Wu F, Zikusoka M, Trindade A, Dassopoulos T, Harris ML (2008). MicroRNAs are differentially expressed in ulcerative colitis and alter expression of macrophage inflammatory peptide-2 alpha.. Gastroenterology.

[23] Latronico MV, Catalucci D, Condorelli G (2008). MicroRNA and cardiac pathologies.. Physiol Genomics.

[24] Yu SL, Chen HY, Chang GC, Chen CY, Chen HW (2008). MicroRNA signature predicts survival and relapse in lung cancer.. Cancer Cell.

[25] Fontana L, Sorrentino A, Condorelli G, Peschle C (2008). Role of microRNAs in haemopoiesis, heart hypertrophy and cancer.. Biochem Soc Trans.

[26] Eder M, Scherr M (2005). MicroRNA and Lung cancer.. N Eng J Med.

[27] Foekens JA, Sieuwerts AM, Smid M, Look MP, de Weerd V (2008). Four microRNAs associated with aggressiveness of lymph node-negative, estrogen receptor positive human breast cancer.. Proc Nat Acad Sci USA.

[28] Neely LA, Rieger-Christ KM, Neto BS, Eroshkin A, Garver J (2008). A microRNA expression ratio defining the invasive phenotype in bladder tumors..

[29] Ozen M, Creighton CJ, Ozdemir M, Ittmann M (2008). Widespread deregulation of microRNA expression in human prostate cancer.. Oncogene.

[30] Sethupathy P, Collins FS (2008). MicroRNA target site polymorphisms and Human disease.. Trends Genet.

[31] Smith DD, Saetrom P, Sneve O, Lundberg C, Rivas GE (2008). Meta analysis of breast cancer microarray studies in conjunction with conserved cis-elements suggest patterns for coordinate regulation.. BMC Bioinformatics.

[32] Verghese ET, Hanby AM, Speirs V, Hughes TA (2008). Small is beautiful: microRNAs and breast cancer-where are we now?. J Pathol.

[33] Ambs S, Prueitt RL, Yi M, Hudson RS, Howe TM (2008). Genomic Profiling of MicroRNA and Messenger RNA Reveals Deregulated MicroRNA Expression in Prostate Cancer.. Cancer Res.

[34] Bloomston M, Frankel WL, Petrocca F (2007). MicroRNA Expression Patterns to Differentiate Pancreatic Adenocarcinoma From Normal Pancreas and Chronic Pancreatitis.. JAMA.

[35] Guo Y, Chen Z, Zhang L, Zhou F, Shi S (2008). Distinctive microRNA profiles relating to patient survival in esophageal squamous cell carcinoma.. Cancer Res.

[36] Laios A, O'Toole S, Flavin R, Martin C, Kelly L (2008). Potential role of miR-9 and miR-223 in recurrent ovarian cancer.. Molecular Cancer.

[37] Wang G, Wang Y, Feng W, Wang X, Yang JY (2008). Transcription factor and microRNA regulation in androgen dependent and independent prostate cancer cells.. Biomed genomics.

[38] Xi Y, Nakajima G, Gavin E, Morris CG, Kudo K (2007). Systematic analysis of microRNA expression of RNA extracted from fresh frozen and formalin-fixed paraffin embedded samples.. RNA.

[39] Chen X, Ba Y, Ma L, Cai X (2008). Characterization of microRNAs in serum: a novel class of biomarkers for diagnosis of cancer and other diseases.. Cell Research.

[40] Gilad S, Meiri E, Yogev Y, Benjamin S, Lebanony D (2008). Serum MicroRNAs are promising biomarkers.. PLoS ONE.

[41] Shih KK, Levine DA (2008). Exosomal microRNAs step into the biomarker arena.. Gynecol Oncol.

[42] Chim SS, Shing TK, Hung EC, Leung TY, Lau TK (2008). Detection and characterization of potential microRNAs in maternal plasma.. Clin Chem.

[43] Santa Lucia J, Hicks D (2004). The thermodynamics of DNA structural motifs.. Ann Rev Biophys Biomol Struct.

[44] Schetter AJ, Leung SY, Sohn JJ, Zanetti KA, Bowman ED, Yanaihara N, Yuen ST, Chan TL, Kwong DL, Au GK, Liu CG, Calin GA, Croce CM, Harris CC (2008). MicroRNA expression profiles associated with prognosis and therapeutic outcome in colon adenocarcinoma.. JAMA.

[45] Monzo M, Navarro A, Bandres E, Artells R, Moreno I, Gel B, Ibeas R, Moreno J, Martinez F, Diaz T, Martinez A, Balagué O, Garcia-Foncillas J (2008). Overlapping expression of microRNAs in human embryonic colon and colorectal cancer.. Cell Res.

[46] Tong AW, Fulgham P, Jay C, Chen P, Khalil I, Liu S, Senzer N, Eklund AC, Han J, Nemunaitis J (2009). MicroRNA profile analysis of human prostate cancers.. Cancer Gene Ther.

[47] Porkka KP, Pfeiffer MJ, Waltering KK, Vessella RL, Tammela TL, Visakorpi T (2007). MicroRNA expression profiling in prostate cancer.. Cancer Res.

[48] Wong TS, Liu XB, Wong BY, Ng RW, Yuen AP, Wei WI (2008). Mature miR-184 as Potential Oncogenic microRNA of Squamous Cell Carcinoma of Tongue.. Clin Cancer Res.

[49] Ghindilis AL, Smith MW, Schwarzkopf KR, Roth KM, Peyvan K (2007). CombiMatrix oligonucleotide arrays: genotyping and gene expression assays employing electrochemical detection.. Biosens Bioelectron.

